# Self-Templating Synthesis of N/P/Fe Co-Doped 3D Porous Carbon for Oxygen Reduction Reaction Electrocatalysts in Alkaline Media

**DOI:** 10.3390/nano12122106

**Published:** 2022-06-19

**Authors:** Yan Rong, Siping Huang

**Affiliations:** 1 College of Resources & Environment and Historical Culture, Xianyang Normal University, 43 Wenlin Road, Weicheng District, Xianyang 712000, China; 2 College of Chemistry and Chemical Engineering, Xianyang Normal University, 43 Wenlin Road, Weicheng District, Xianyang 712000, China; huangsiping1971@163.com

**Keywords:** N/P/Fe co-doped carbon, self-templating synthesis, 3D porous structure, oxygen reduction reaction electrocatalysts

## Abstract

The development of low-cost, highly active, and stable oxygen reduction reaction (ORR) catalysts is of great importance for practical applications in numerous energy conversion devices. Herein, iron/nitrogen/phosphorus co-doped carbon electrocatalysts (NPFe-C) with multistage porous structure were synthesized by the self-template method using melamine, phytic acid and ferric trichloride as precursors. In an alkaline system, the ORR half-wave potential is 0.867 V (vs. RHE), comparable to that of platinum-based catalysts. It is noteworthy that NPFe-C performs better than the commercial Pt/C catalyst in terms of power density and specific capacity. Its unique structure and the feature of heteroatom doping endow the catalyst with higher mass transfer ability and abundant available active sites, and the improved performance can be attributed to the following aspects: (1) Fe-, N-, and P triple doping created abundant active sites, contributing to the higher intrinsic activity of catalysts. (2) Phytic acid was crosslinked with melamine to form hydrogel, and its carbonized products have high specific surface area, which is beneficial for a large number of active sites to be exposed at the reaction interface. (3) The porous three-dimensional carbon network facilitates the transfer of reactants/intermediates/products and electric charge. Therefore, Fe/N/P Co-doped 3D porous carbon materials prepared by a facile and scalable pyrolysis route exhibit potential in the field of energy conversion/storage.

## 1. Introduction

Oxygen reduction reaction (ORR) kinetics pose a major challenge to the development of energy conversion devices, especially metal-air batteries and fuel cells [1,2,3,4]. Platinum based noble metal catalysts are recognized as the best catalysts for ORR. However, its large-scale commercial application is greatly limited by scarce resources, high cost and poor durability [5,6,7]. Therefore, there is an urgent need to develop non-precious metal-based catalysts apply earth-rich materials [8,9]. Among existing non-noble metal based catalysts, the incorporation of heteroatoms into carbon based materials can change the charge distribution (charge distribution or spin density redistribution), which can introduce abundant active sites and thus enhance the adsorption of and reduction in oxygen [10,11,12] Researchers have confirmed that the structure design of carbon-based materials is of great significance to the improvement of properties [6,13,14,15]. In addition, researchers have found that active species such as transition metals (Mn, Fe, Co, Ni, Cu, etc.) and heteroatoms (B, N, F, P, S, etc.) in carbon-based nanomaterials have synergistic effects to change the topical work function of materials, resulting in enhanced ORR performance [16,17].

Generally, the catalytic performance of carbon-based doped catalysts depends on the specific surface area and pore structure, the composition, content, and distribution of doped elements [18,19,20,21]. Conventional synthetic techniques are mainly obtained by porous templates at high carbonization temperatures [22,23]. However, template-assisted methods often require additional complex processes to synthesize and remove templates (for example, silica, polystyrene spheres or metal oxides), during which toxic reagents may be introduced, causing the catalyst to be poisoned. Therefore, it is of great importance to develop a simple process to prepare efficient carbon-based catalysts with good doping conditions and three-dimensional porous structure for ORR catalysis [6,10,24,25,26,27].

**Figure 1:**
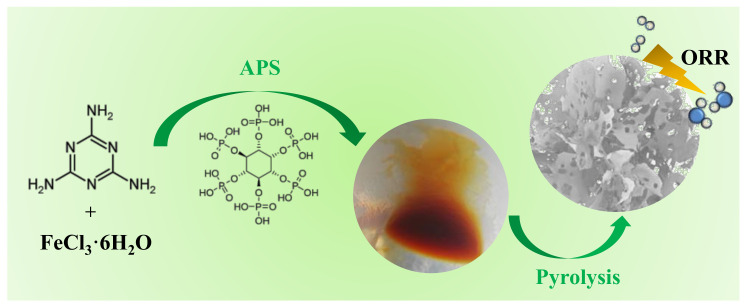
Schematic diagram of the preparation of the NPFe-C.

Herein, the 3D N/P/Fe co-doped material with reasonable pore size and heteroatom distribution was prepared by self-template assisted preparation. The porous structure and surface area were constructed to enrich the active sites exposed to the catalytic reaction interface [28]. Melamine coordinates with Fe and crosslinks with phytic acid (PA) to form a hydrogel. In the pyrolysis process, the decomposition of melamine with PA will generate a large number of micropores and small-sized mesopores, so that the doped atoms are exposed at the three-phase boundary, generating a large number of available active sites (Figure 1). It is expected to promote the formation of carbon porous structures, increase the surface area, and expose more active sites. Three-dimensional interconnected porous networks contribute to the diffusion and transport of substances involved in the reaction (such as O_2_ and H_2_O) in the process of oxygen reduction. Benefiting from the well controlled distribution and content of N, P, and Fe atoms, as well as the synergistic effect of three-dimensional hierarchical structures, the NPFe-C exhibit much better ORR catalytic activity and durability than the currently advanced Pt/C catalysts. In addition, compared with the traditional templating method, PA cross mixing with melamine hydrogel serves as a self sacrificial template, avoiding the unfavorable process of template removal.

## 2. Materials and Methods

### 2.1. Reagents and Chemicals

Iron trichloride (FeCl_3_), melamine (C_3_H_6_N_6_), phytic acid (C_6_HOP_6_) were purchased from Sinopharm Chemical Reagent Co., Ltd. (Shanghai, China). Commercial 20 wt% Pt/C elelctrocatalyst was supplied by E-TEK, Inc (Shenzhen, China). Potassium hydroxide (KOH) and hydrochloric acid (HCl) were supplied by Aladdin Reagent Co., Ltd. (Shanghai, China).

### 2.2. Preparation of NPFe-C

A total of 0.5 g of melamine and 0.1 g of FeCl_3_ were added in 200 mL water. Then, 0.1 mL of PA was added in the mixture. After stirring for 3 h, the gel product was recovered, dried, and carbonized at 800 °C for 2 h under a N_2_ flow with a heating rate of 1 °C/min. Finally, the NPFe-C were obtained after immersed in 0.5 M HCl solution for 3 h. For comparison, the Fe-free catalyst (NP-C) was synthesized by similar processes.

### 2.3. Physical Characterization

The crystalline structure, morphology and surface composition of the sample were physically characterized by X-ray diffraction (XRD), Raman Spectrometer, scan electron microscopy (SEM) equipped with energy-dispersive spectrometer (EDS), transmission electron microscopy (TEM), and X-ray photoelectron spectroscopy (XPS). The surface area and pore volume of the samples were measured on a physical adsorption instrument.

### 2.4. Electrochemical Measurement

The electrochemical experiments were carried out on a computer-controlled CHI 760E electrochemical workstation. The standard 3-electrode system was used at 30 °C, a rotating disk electrode (RDE, 0.196 cm^2^) or a rotating ring disk electrode (RRDE, 0.2475 cm^2^) coated with the electrocatalyst was used as working electrode, a carbon rod served as counter electrode, and a saturated calomel electrode acted as reference electrode. The electrocatalyst ink was obtained by mixing 10 mg of electrocatalyst and 5 mL of water/ethanol/Nafion solution, and sonicating for 60 min. Then, 10 μL electrocatalyst ink was dropped onto the polished glassy carbon electrode surface and dried at room temperature. The catalyst loading is 0.10 mg cm^−2^. The CV curve and LSV curve were recorded in the N_2_/O_2_-saturated 0.1 M KOH solution. The rotating speed and potential sweep rate were set to 1600 rpm and 10 mV s^−1^, respectively [29].

### 2.5. Zn−Air Battery Measurements

The electrochemical properties of the catalysts were evaluated in the simulated Zn-air battery devices. Polarization curves and galvanostatic discharge tests were carried out on a CHI 760E electrochemical workstation and LAND testing system, respectively. The as prepared electrocatalyst ink was coated on PTFE treated carbon pentafiber paper as cathode, and zinc foil and 6 M KOH were used as anode and electrolyte [25].

## 3. Results

### 3.1. Characterization of NPFe-C

**Figure 2:**
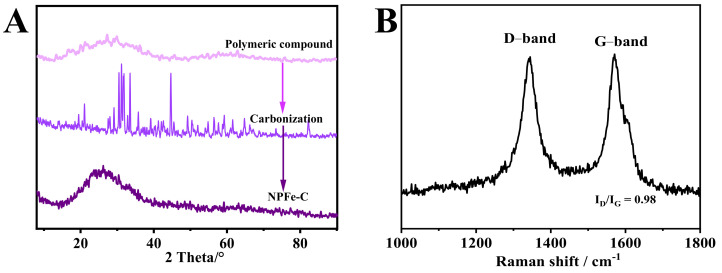
(**A**) XRD patterns and (**B**) Raman spectrum of the NPFe-C.

The samples at different synthesis stages were characterized by XRD (Figure 2A). The samples before carbonization had wide C (002) peaks at 25°. After carbonization at 800 °C, the sample shows 3 sets of X-ray diffraction peaks, which are Fe (PDF#52-0513), Fe (PDF#65-4899), Fe_3_O_4_ (JCPDS No.11-0614) and Fe_3_P (PDF#19-0617). For NPFe-C, there are a few weak and widened diffraction peaks, which may be due to the existence of trace iron-based nanocrystals. In addition, 2 broad characteristic peaks can be observed after carbonization of polymer samples, which correspond to the (002) and (100)/(101) crystal planes of carbon, about 25° and 42°, respectively [30]. The corresponding (002) diffracted graphite plane indicates a highly crystalline structure. Raman spectroscopy (Figure 2B) confirms this assertion by using the intensity ratios of D to G bands (I_D_/I_G_) caused by Raman vibrations of disordered (sp3) and ordered (sp2) carbon, respectively. I_D_/I_G_ of D-band (1337 cm^−1^) and G-band (1587 cm^−1^) with relative peak strength of NPFe-C is 0.93, it indicates that the material has both amorphous carbon and graphitized carbon with high degree of graphitization. In general, carbon-based electrocatalysts with a high graphitization degree increase the electrical conductivity, thus effectively improving the activity of the ORR [31].

**Figure 3:**
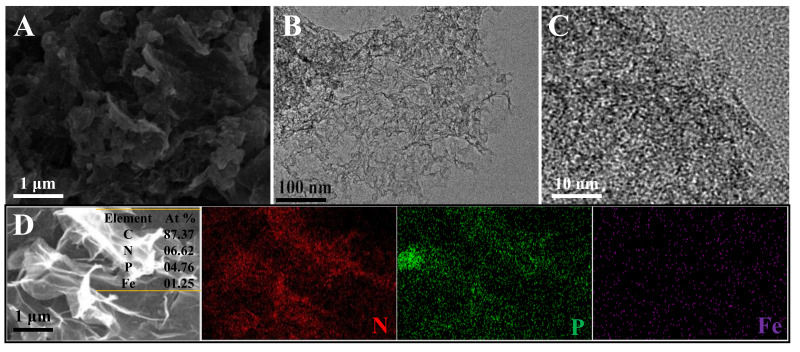
(**A**) SEM images, (**B**) TEM images, (**C**) HRTEM image and (**D**) elemental mappingof the NPFe-C. The insets are the corresponding EDS data.

NPFe-C was analyzed by SEM (Figure 3A) and TEM (Figure 3B), and the pyrolyzed NPFe-C catalysts exhibited typical interconnected 3D macroporous networks. High resolution TEM (HRTEM) images of the NPFe-C reveal that the material is not dense and contains numerous pores (Figure 3C). The mapping results show that N, Fe and P elements are evenly distributed in the three-dimensional carbon network (Figure 3D).

**Figure 4:**
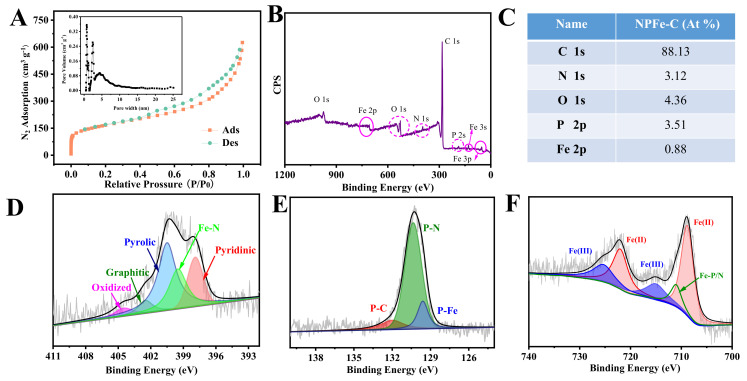
(**A**) N_2_ adsorption–desorption isotherm curve (inset: pore size distribution) of NPFe-C, (**B**,**C**) XPS spectrum and the corresponding data of the Polymeric compound, the Carbonization and the NPFe-C, (**D**–**F**) high-resolution XPS spectra of N 1s, P 2p, Fe 2p of NPFe-C.

To further investigate the pore structure of the materials, the pore structure of NPFe-C was characterized by N_2_ adsorption desorption isotherms (Figure 4A). The isothermal curves showed a type I/IV mixing shape. The 2 steep adsorption processes occurred at low (<0.05) and high pressures (>0.9) and showed the coexistence of micropores, mesopores, and macropores. The specific surface area was calculated to be 774.5 m^2^ g^−1^. The simulation results based on QSDFT model show that the micropore size in NPFe-C is mainly 0.9 nm and the mesoporous size is widely distributed in the range of 2–35 nm. The micro/mesopores are formed during carbonization, in which the polymer is pyrolyzed to generate volatile gas, forming porous carbon. The hierarchical pore structure may facilitate the diffusion of substances during the ORR process. The larger BET surface area and abundant microporous structures is conducive to expose the abundant ORR active sites to the reaction interface and promote the promotion of the activity of NPFe-C catalysts. In addition, constructing a reasonable 3D microporous structure can provide efficient mass transport channels for reactants, intermediates, and products. All of these features are useful for improving ORR performance.

The surface composition of NPFe-C was further analyzed by X-ray photoelectron spectroscopy (XPS). The presence of C, N, O, P, Fe in the doped carbon materials was confirmed by XPS survey spectra (Figure 4B), indicating the successful introduction of heteroatoms into the carbon substrate. The N, P, and Fe contents in NPFe-C were 2.12, 1.24, and 0.31 at%, respectively (Figure 4C). The high-resolution N1s spectra of the heteroatom doped catalysts (Figure 4D) are classified into 5 types, including pyridinic-N (398.2 eV, 26.7%), Fe-Nx (399.5 eV, 19.8%), pyrrolic-N (401.1 eV, 35.6%), graphitic-N (402.4 eV, 9.2%), and oxidized N (404.7 eV, 8.7%), some studies have demonstrated that pyridinic-N, Fe-Nx and graphitic-N are active sites for the ORR [32,33,34,35]. Some researchers have proposed that the N atom can reduce the band gap and increase the charge mobility of graphite lattices. Quantum chemical calculations show that the N-containing group at the edge has the highest charge mobility. In addition, these changes in the carbon band structure ultimately reduce the electronic work function at the interface, which has an important impact on the ORR performance [6,36]. The P 2p high resolution spectrum has two characteristic peaks at 132.9 eV and 131.2 eV, which are assigned to P-C and P-O species respectively. Doped P mostly exists in P-C (Figure 4E). Because the electronegativity of P (2.19) is lower than C (2.55), P atom with positive charge will be induced and new ORR active sites may be generated in alkaline medium [2]. Moreover, the presence of phosphorus atoms in P-O is beneficial for enhancing the electronegativity of the O atom, thereby promoting the charge delocalization of nearby C atoms, increasing the adsorption on O_2_ [37,38,39]. Moreover, P atom doping in the carbon lattice induces distortion of its structure, and generates edge sites, which further enhance its electrochemical activity [40]. As identified by XPS, the high-resolution Fe 2p spectrum (Figure 4F) has obvious peaks at 725.7 eV and 721.3 eV, which are the Fe 2p 1/2 of Fe (III) and Fe (II), respectively. The peak at 709.2 eV is Fe 2p 3/2, while the peak at 716.3 eV is probably a satellite peak. According to previous studies, Fe sites are favorable for the adsorption of O_2_ and oxygenated intermediates, thereby significantly promoting the activity of ORR [41,42,43].

### 3.2. ORR Performance and the Zn-Air Battery Tests of NPFe-C

**Figure 5:**
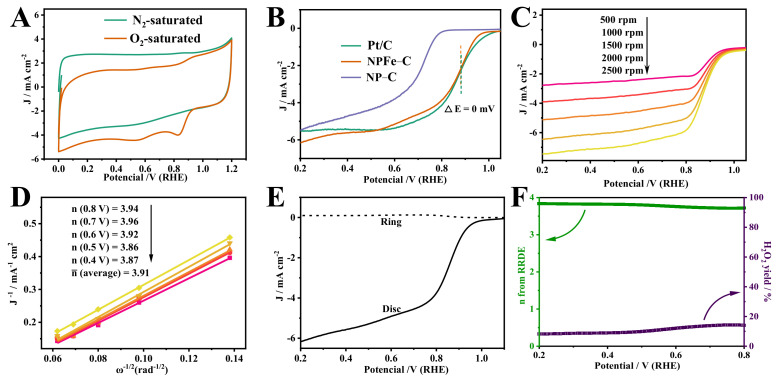
(**A**) CV curves of NPFe-C in an N_2_- and O_2_-saturated 0.1 M KOH solution. (**B**) LSV of NPFe-C, NP-C and commercial Pt/C in an O_2_-saturated 0.1 M KOH solution (sweep rate: 10 mV s^−1^; electrode rotation speed: 1600 rpm). (**C**) LSV of NPFe-C at different rotation rates. (**D**) Koutecky-Levich of NPFe-C. (**E**,**F**) Rotating ring-disk electrode voltammograms obtained at 1600 rpm in O_2_ saturated 0.1 M KOH solutions.

The ORR activity of NPFe-C was first measured by cyclic voltammetry (CV) in 0.1 M KOH electrolyte saturated with N_2_/O_2_ (Figure 5A). In oxygen saturated electrolyte, a cathode peak was observed at 0.81 V. In contrast, such cathode peaks were not found in N_2_-saturated electrolytes. This indicates that NPFe-C has significant electrocatalytic activity for ORR. The ORR performance of NPFe-C electrocatalysts was investigated by rotating disk electrode tests (Figure 5B). The polarization curves show that the NPFe-C catalysts exhibit the most superior electrocatalytic activity for half wave potential of 0.867 V compared with NP-C (E_1/2_ = 0.712 V), even comparable to that of the commercial catalyst. We observed that the annealing temperature significantly affected the ORR activity of N/P/Fe-C (Figure S1A). According to the E_1/2_ value of ORR and the limiting current density, the catalyst with the best performance was obtained at 800 °C. In addition, the formation of N/P/Fe-C and the ORR performance were significantly affected by different ratio of reagents. The ORR activity of the samples with the ratio of melamine to phytic acid of 0.5 g:0.1 mL is significantly better than that of 0.5 g:0.04 mL, 0.5 g:0.06 mL, 0.5 g:0.15 mL and 0.5 g:0.2 mL (Figure S1B). When changing the amount of FeCl_3_, the ORR activity tends to increase with the amount of FeCl_3_ added, but the hydrogel can not be obtained when the amount is more than 0.12 g (Figure S1C).

To further investigate the electron transfer process of NPFe-C catalysts, the ORR polarization curves at different rotating speeds were recorded in saturated 0.1 M KOH solution. The kinetic parameters were analyzed using the koutecky Levich (K-L) equation (Figure 5C,D), which revealed that the electron transfer number was close to 4, indicating high selectivity for the complete four electron oxygen reduction reaction. The selectivity of ORR was then determined by using the rotating ring disc electrode (RRDE) (Figure 5E,F). The disc current density (I_D_) sharply increased over 0.8 V. Similar to what RDE observed, the disc current (I_R_) was negligible in the range of 0.2–0.8 V, corresponding to a lower hydrogen peroxide yield of 7–12%. Therefore, in this potential range, the value of n is about 3.88, which is consistent with the results of RDE.

**Figure 6:**
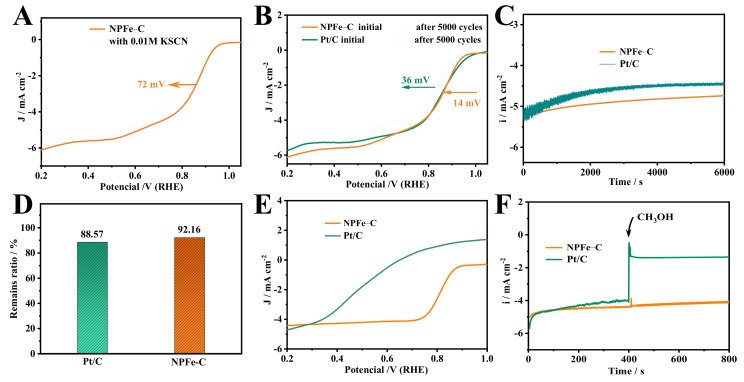
(**A**) ORR polarization curves of Fe-NCCs electrocatalysts in 0.1 M KOH + 0.01 M KSCN solution, (**B**) ORR curves before and after 5000 CV cycles (CV potential range: 0 V–1.2 V, RHE), (**C**,**D**) Chronoamperometric curves and the corresponding data of NPFe-C and Pt/C electrocatalyst, (**E**) ORR polarization curves of Fe-NCCs in 0.1 M KOH + 0.2 M methanol solution, (**F**) Chronoamperometric curves of the Pt/C electrocatalyst and NPFe-C before and after adding 1 M methanol.

In order to further explore the active sites of NPFe-C, the method of SCN^-^ poisoning catalyst was adopted. It is well known that SCN^−^ poisons the active site of Fe-N/Fe-P and inactivates its active site because of the strong action of SCN^−^ ions on Fe. As shown in Figure 6A, after the introduction of 0.1 M KSCN, E_1/2_ was negatively shifted by 72 mV, consistent with the results reported in the literature [43,44,45]. Therefore, the presence of Fe-N/Fe-P species is essential to provide high performance. Moreover, the E_1/2_ of the catalyst still reaches 0.78 V after the Fe-N/Fe-P species is toxified, indicating that Fe-N is not the only active site and part of the activity of the catalyst originates from the doping of heteroatoms. Therefore, it can be concluded that both Fe-N/Fe-P species and doped nitrogen atoms contribute to the improvement of ORR performance of NPFe-C. In addition, the electrical conductivity of the materials significantly affects the catalytic performance. The conductivity of catalysts is evaluated by EIS test. The charge transfer resistance (Rct = 193.62 Ω) of NPFe-C is similar to that of Pt/C (Rct = 137.75 Ω) (Figure S2). Since the electrical conductivity of carbon materials is related to their graphitization degree, combined with Raman measurements, it is concluded that the graphitization degree of N/P/Fe-C contributes to the kinetic improvement of ORR.

In addition to its high catalytic activity, the long-term stability of the electrocatalysts must also be considered. The stability of NPFe-C was evaluated using cycling experiments (Figure 6B) and chronoamperometry (Figure 6C). After 5000 cycles, the E_1/2_ of NPFe-C only decreased by 14 mV. In contrast, the E_1/2_ of the Pt/C electrocatalyst is negatively shifted by 36 mV. The i-t curves show that the NPFe-C decays more slowly than the Pt/C electrocatalyst at 0.8 V (Figure 6C). Among them, NPFe-C maintained 92.16% of the initial current value after 6000 s; this was much better than the commercial Pt/C electrocatalyst (88.57%). These results demonstrate that the NPFe-C catalysts exhibit remarkable electrocatalytic activity and long-lasting stability in alkaline medium, serving as a cost-effective electrocatalyst for ORR.

Over the past 30 years, direct methanol fuel cells (DMFCs) have emerged as a highly promising energy conversion device. In the DMFC system, the methanol fuel of the anode causes the cathodic Pt/C electrocatalyst to generate mixed potentials and CO poisoning. Therefore, the cathode electrocatalyst with high ORR selectivity has an important promoting effect on the commercialization of DMFCs. To examine the ORR selectivity of the cathode electrocatalyst, the ORR performance of the NPFe-C and commercial Pt/C in the presence of methanol was investigated (Figure 5E,F) [29]. As shown in Figure 6E, the E_1/2_ value of the ORR for NPFe-C is more positive than that of commercial Pt/C in 0.1 M KOH + 0.2 M methanol solution. The i-t curves show that the ORR current of NPFe-C during the first 400 s is comparable to that of the Pt/C electrocatalyst in the absence of methanol, and after 1 M methanol was injected into the electrolyte at 400 s, there was no obvious decrease in the current of NPFe-C but not that of the Pt/C electrocatalyst. Therefore, with the addition of 1 M methanol, the electrocatalytic activity of NPFe-C for ORR is much higher than that of Pt/C electrocatalysts, indicating the high selectivity of NPFe-C for ORR.

**Figure 7:**
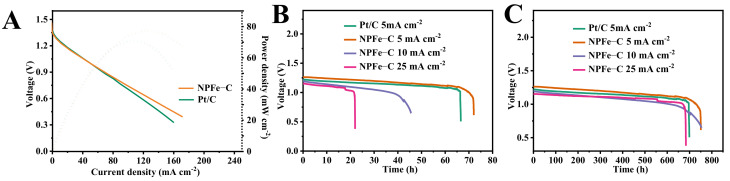
(**A**) Polarization and power-density curves, (**B**) galvanostatic discharge curves; (**C**) specific capacities at various current densities (5, 10, and 25 mA cm^−2^) of the Zn-air batteries using NPFe-C and Pt/C as electrocatalysts.

In order to further evaluate the ORR activity of NPFe-C catalyst under actual battery operating conditions, NPFe-C and Pt/C were assembled to fabricate Zn-air batteries, the 6 M KOH solution was used as the electrolyte. As shown in Figure 7A, the Zn-air battery with NPFe-C as catalyst exhibits higher discharge current and power density than that with Pt/C. The maximum power density of NPFe-C catalyst is 79.6 mWcm^−2^, which is significantly higher than that of commercialized Pt/C (68.5 mWcm^−2^). In addition, typical galvanostatic discharge tests were performed at different current densities (Figure 7B), which revealed that the NPFe-C based cells exhibited a slightly higher voltage (1.23 V) and a longer discharge time (74 h) compared to the Pt/C electrocatalyst at 5 mA cm^−2^ current density (1.22 V, 68 h). When normalized to the mass of consumed Zn (Figure 7C), the calculated cell specific capacity is 752 mAh g^−1^ at 5 mA cm^−2^ for the NPFe-C catalyzed air cathode, superior to that of commercialized Pt/C (707 mAh g^−1^). In particular, the NPFe-C still exhibits excellent electrocatalytic performance for Zn-air batteries under high current conditions.

## 4. Conclusions

In summary, N-, P-, and Fe triple doped three-dimensional nanoporous carbon materials synthesized by a simple self templating strategy, with large specific surface area, optimal porous structure, and well-designed distribution of doped atoms, effectively enhance the number of active sites and mass transfer efficiency. Compared with commercial Pt/C catalysts, NPFe-C catalysts show better electrocatalytic activity and long-term durability for ORR. Assembled in the Zn-air cell, the catalyst exhibits superior power density and specific capacity over the Pt/C catalyst. These results indicate that NPFe-C as ORR electrocatalysts can be used as potential substitutes for precious metal catalysts in energy conversion and storage devices.

## Data Availability

Not applicable.

## Supplementary Materials

The following supporting information can be downloaded at: https://www.mdpi.com/article/10.3390/nano12122106/s1, Figure S1: ORR LSV curves of a series of NPFe-Cs samples with (A) different annealing temperature; (B) different ratio of melamine to phytic acid and (C) different ratio of melamine to FeCl_3_.; Figure S2: EIS curves of the N/P/Fe-C and Pt/C in 0.1 M KOH electrolyte. Potential: at open circuit potential from 0.01 to 100,000 Hz with a modulation amplitude: 10 mV.
