# Supplementary material for: Self-Templating Synthesis of N/P/Fe Co-Doped 3D Porous Carbon for Oxygen Reduction Reaction Electrocatalysts in Alkaline Media
Source: Nanomaterials (Basel). 2022 Jun 19;12(12):2106. doi: 10.3390/nano12122106 (PMC9228732; doi:10.3390/nano12122106)
Supplement: Supplementary file 1 [file nanomaterials-12-02106-s001.zip › nanomaterials-1766625-supplementary.pdf]

Supplementary Materials

Self-Templating Synthesis of N/P/Fe co-Doped 3D Porous Carbon for Oxygen Reduction Reaction Electrocatalysts in Alkaline Media

Yan Rong *,[1] and Siping Huang [2]

[1] College of Resources & Environment and Historical Culture, Xianyang Normal University, 43 Wenlin Road, Weicheng District, Xianyang 712000, China; allenry@126.com
[2] College of Chemistry and Chemical Engineering, Xianyang Normal University, 43 Wenlin Road, Weicheng District, Xianyang 712000, China; huangsiping1971@163.com
* Correspondence: allenry@126.com

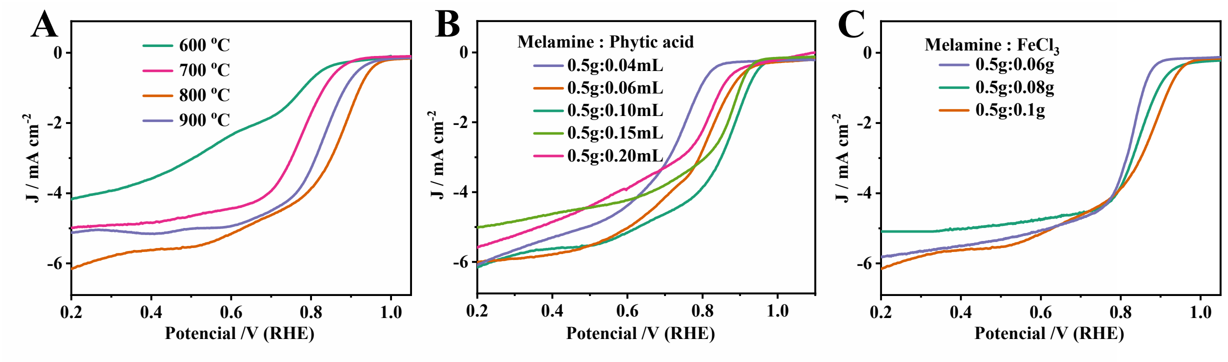

Figure S1 ORR LSV curves of A series of NPFe-Cs samples with (**A**) different annealing temperature; (**B**) different ratio of melamine to phytic acid and (**C**) different ratio of melamine to $FeCl_3$.

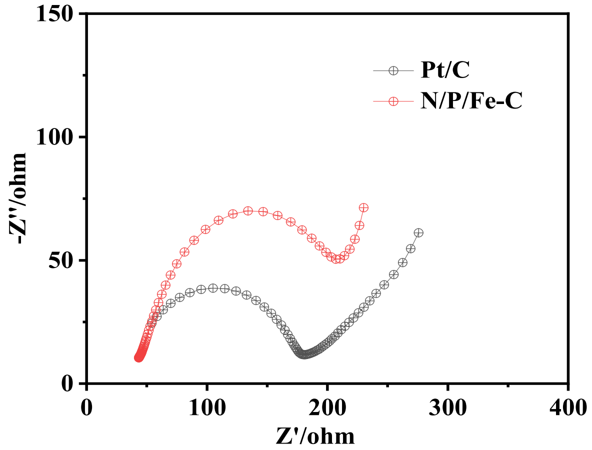

Figure S2. EIS curves of the N/P/Fe-C and Pt/C in 0.1 M KOH electrolyte. Potential: at open circuit potential from 0.01 to 100,000 Hz with a modulation amplitude: 10 mV.
